# Evaluating the Impact of a Sustainability Education Intervention on Emergency Medicine Residents

**DOI:** 10.5811/westjem.56998

**Published:** 2026-08-10

**Authors:** Atieh D. Ashkezari, Christina Bogdani, Xueqing Huang, Michael Gindi

**Affiliations:** * New York Institute of Technology College of Osteopathic Medicine, Northern Boulevard, Old Westbury, New York; † New York Institute of Technology College of Engineering and Computing Sciences, Northern Boulevard, Old Westbury, New York

## Abstract

**Introduction:**

Healthcare is a significant contributor to global greenhouse gas emissions, with emergency departments being among the most resource-intensive settings. Despite increasing awareness of climate change and its health implications, structured sustainability education remains lacking in emergency medicine graduate medical education training. We evaluated the impact of a brief, educational intervention on emergency medicine (EM) residents’ knowledge, attitudes, and self-reported behaviors related to sustainability.

**Methods:**

We conducted a prospective pre/post interventional study at two community-based EM residency programs. Emergency medicine residents completed a baseline survey assessing sustainability-related knowledge, attitudes, and self-reported behaviors, followed by a video on sustainable clinical practice and a peer-reviewed paper on the environmental impact of inhalers. Immediate postcourse and one-month follow-up surveys were administered to evaluate short- and intermediate-term changes. Our primary outcome measure was change in sustainability-related knowledge scores. Secondary outcomes included changes in attitudes toward sustainability and self-reported sustainability-related clinical behaviors. Survey responses were analyzed using descriptive statistics, paired *t* tests and repeated-measures analysis of variance, as appropriate.

**Results:**

Of 41 eligible EM residents across two residency programs, 34 completed the baseline survey (82.9%), 28 completed the immediate post-intervention survey (68.3%), and 30 completed the one-month follow-up survey (73.2%). Knowledge scores at baseline were 55.0% and increased to 68.0% immediately after the intervention (*P* < .001). At one month, knowledge scores remained higher than baseline (65.0%) (*P* = .003). Attitudes improved significantly immediately post-intervention (*P* = .001), despite already neutral-to-positive baseline levels. Two self-reported behavior items improved at one month: preferential prescribing of dry-powder inhalers, which were described in the educational materials as having less of an environmental impact than aerosolized inhalers (12.0% to 37.0%, *P* < .050) and self-reported efforts to reduce unnecessary waste (56.0% to 87.0%, *P* < .050), with overall self-reported behavior change (*P* = .001). Nebulizer-related prescribing did not significantly change. The open-ended item showed similar thematic patterns at both baseline and after one month, with most residents describing simple waste-reduction actions (eg, minimizing disposables), and no significant shift in qualitative categories.

**Discussion and Conclusion:**

A brief, structured sustainability module delivered via a video format was feasible and well-received by EM residents. Preliminary data suggest improvements in knowledge, attitude, and self-reported behaviors, highlighting the potential for scalable sustainability education within graduate medical training. However, these findings should be interpreted in the context of a small sample size and self-reported outcomes.

## INTRODUCTION

The healthcare sector accounts for nearly 9% of total greenhouse gas emissions in the United States, with hospitals and clinical care settings representing major contributors due to substantial energy demands and consumption, extensive use of supplies, and significant waste generation.^1^,^2^ Among these settings, emergency departments (ED) are particularly resource intensive. They operate continuously and rely heavily on disposable supplies and high-emission clinical processes such as diagnostic imaging, all of which contribute substantially to healthcare-related emissions.^3^,^4^ While the relationship between healthcare delivery and climate change has received growing attention in recent years, formalized sustainability training remains limited across undergraduate, graduate, and continuing medical education levels.^5^

Environmental sustainability in healthcare is increasingly being recognized as a core competency for health professionals.^6^ Previous studies have highlighted that physicians often lack formal training on how clinical decision-making influences environmental outcomes, including emissions associated with pharmaceuticals, single-use devices, and over-used imaging.^7^ Quality improvement projects in anesthesia, such as Project SPRUCE, have shown that providing educational modules, clinical decision support, and real-time feedback can lead to substantial reductions in emissions from inhaled anesthetics, with educational interventions being a key driver of sustained practice change.^8^ However, few published studies have evaluated how sustainability training can be effectively integrated into the fast-paced, high-acuity environment of emergency medicine. A recent perspective from the United Kingdom highlighted growing momentum for sustainable healthcare within EM and outlined actionable strategies to improve environmental practices in EDs, including reducing the use of inhaled agents with high potential for global warming, optimizing waste segregation, and incorporating telemedicine to limit travel-related emissions.^9^

Despite these developments, a significant gap regarding the integration of formal sustainability training into U.S.-based residency programs remains. To address this gap, in this study we evaluated the feasibility of an educational intervention and its impact on EM residents’ knowledge, attitudes, and self-reported behaviors as related to sustainability—delivered via a brief, video-based educational module and a peer-reviewed paper— aimed to provide foundational knowledge on the environmental impact of clinical care and encourage reflection on sustainable practices in the ED.^10^, ^11^

Residency training represents a particularly high-leverage opportunity, as it shapes long-term clinical decision-making patterns and practice habits.^12^ Introducing sustainability concepts at this stage may facilitate early integration of environmentally conscious practices into routine care.

**Population Health Research Capsule**

What do we already know about this issue?

*Healthcare contributes significantly to greenhouse gas emissions, yet sustainability education in emergency medicine (EM) training remains limited*.

What was the research question?

*Does a brief educational intervention improve EM residents’ sustainability knowledge, attitudes, and behaviors?*

What was the major finding of the study?

*Knowledge improved from 55% to 68% immediately post-intervention (P < .001) and remained higher at one month (65%, P = .003)*.

How does this improve population health?

*Integrating sustainability education into residency training may promote environmentally responsible clinical practices and reduce healthcare-related emissions*.

## METHODS

### Study Design and Setting

This study was a prospective, single-arm educational intervention conducted across two community hospitals within the same hospital system in New York State. Both sites host accredited EM residency programs that share a common academic structure and didactic schedule. The study was designed to evaluate the impact of a structured sustainability education module on EM residents’ knowledge, attitudes, and self-reported behaviors related to environmentally sustainable clinical practice.

### Participants

All postgraduate year (PGY) 1–3 EM residents scheduled for weekly didactic sessions during the study period were invited to participate. Participation was voluntary, and survey results were anonymous. Only residents present during the assigned educational session were eligible to complete the surveys. No identifying information was collected at any stage of the study.

### Intervention

The intervention consisted of a 60-minute video module, *Healthcare Sustainability – Transforming Clinical Practice in the Face of Climate Change*, developed by Mass General Hospital and publicly available on YouTube^10^ and an accompanying peer-reviewed paper on the environmental impact of asthma inhalers by Wilkinson and Woodcock.^11^ Together, these resources provide a comprehensive overview of the environmental impact of healthcare delivery, principles of sustainable clinical decision-making, and practical strategies to reduce waste in EM. For both community hospitals, the intervention was delivered in person without additional facilitation or discussion components to ensure consistency across sites.

### Survey Instrument

We developed a structured survey instrument and sent it electronically using REDCap (Research Electronic Data Capture) to assess participants’ knowledge, attitudes, and self-reported behaviors related to environmental sustainability in healthcare. Surveys were distributed at three timepoints: baseline (preintervention); immediate postintervention; and one-month follow-up, with survey content tailored to the intervention’s learning objectives and knowledge-retention assessment at each stage. Each survey began with an eligibility and consent confirmation, requiring participants to verify that they were ≥ 18 years of age and voluntarily agreed to participate. All responses were collected anonymously.

### Survey Development and Rationale

The survey was developed to target high-yield concepts in clinical sustainability that align with the content of the educational module and with evidence-based practices in healthcare environmental impact. We selected knowledge items to address common areas of limited awareness among clinicians, including the environmental consequences of personal protective equipment selection (Q1); inhaler propellants and prescribing decisions (Q2, Q4–Q6, Q9); sterilization practices and device reprocessing (Q3, Q12, Q13); and the effects of low-value care such as unnecessary imaging or routine testing (Q7, Q14).^7^ Several questions (Q10, Q15–Q17) were included to contextualize the scale of healthcare-related emissions using widely recognized benchmarks, including the Scope 1–3 framework for measuring an organization’s greenhouse gas emissions in which supply-chain emissions (Scope 3) represent the dominant contributor to healthcare’s carbon footprint.^13^

Certain items (eg, inhaler-related questions) were simplified for educational purposes and may not have fully captured nuances such as differences between dry-powder and metered-dose inhalers. Attitude items assessed perceived relevance, feasibility, and educational value of sustainability in EM, while behavior items captured prescribing choices, self-reported waste-reduction practices, and personal sustainability strategies. All items underwent content review by faculty members with expertise in EM and sustainability to ensure clarity and alignment with the intervention’s learning objectives.

### Baseline Survey

The baseline survey collected demographics (PGY level, sex, additional academic degrees, and age), 17 knowledge items, eight attitude items, and four behavior items, including one open-ended question regarding personal sustainability practices.

### Immediate Postintervention Survey

The immediate postintervention survey repeated the knowledge and attitude items verbatim from the baseline survey to measure short-term learning gains and attitudinal shifts following the educational module. Behavioral questions were excluded at this stage as meaningful changes in clinical practice behavior require time to develop.

### One-Month Follow-Up Survey

The one-month survey included brief demographic verification (PGY level and sex) to confirm respondent consistency and repeated the knowledge and behavior domains to assess retention of learning and translation into self-reported clinical practice changes after one-month postintervention. At one month, behavior items, which reflect the downstream application of attitudes, were prioritized over repeating attitudinal questions.

### Scoring

We calculated knowledge scores as the percentage of correct responses. Attitude items were summarized using mean Likert values per item, and behavioral responses were reported as categorical frequencies. For graphical visualization, Likert responses (1–5) were converted to a normalized 0–1 scale. Open-ended responses were reviewed qualitatively to identify recurring sustainability themes.

### Data Collection

Surveys were distributed and completed through a REDCap link. The baseline survey was administered immediately prior to the video session, the postintervention survey immediately afterward, and the one-month survey distributed via email reminder. Responses were automatically stored in REDCap and exported for analysis.

### Statistical Analysis

The primary outcome was change in sustainability-related knowledge scores across time points. Secondary outcomes included changes-in-attitude composite scores and self-reported sustainability-related behaviors. All analyses were performed in Python v3.11 (Python Software Foundation, Wilmington, DE). Descriptive statistics were used to summarize demographic data and response distributions. Repeated measures analysis of variance was used to evaluate changes in knowledge across the three time points (baseline, immediate, one month), followed by Bonferroni-adjusted paired *t*-tests when the omnibus test was significant. Paired *t*-tests were used to compare attitude scores between baseline and immediate post-intervention. Behavioral outcomes measured at baseline and one month were analyzed using paired *t*-tests and the McNemar test for paired categorical variables. A two-tailed *P* < .050 was considered statistically significant.

Effect sizes (Cohen *d*) were calculated for primary comparisons to provide estimates of magnitude of change.

### Ethical Considerations

This study was reviewed and approved by the institutional review board (IRB-2026-355) and deemed exempt under educational quality-improvement criteria. Because all data were collected anonymously and no patient-level information was used, separate IRB review by the participating EM training sites was not required. All identifying information was removed to maintain double-blinded peer review per journal requirements.

## RESULTS

Of 41 eligible EM residents across two programs, 34 completed the baseline survey (82.9% response rate), 28 completed the immediate postintervention survey (68.3%), and 30 residents completed the one-month follow-up survey (73.2%), reflecting variation in participation across time points due to attendance and follow-up survey completion. Baseline demographic information, including postgraduate year level, sex, and additional academic degrees, is presented in Table 1.

### Knowledge Results

Seventeen multiple-choice and true/false questions assessed residents’ knowledge of sustainability in healthcare. The mean percentage of correct responses increased from 55.0% at baseline to 68.0% immediately postintervention, corresponding to a large effect size (Cohen *d* =. 1.048) (Figure 1). At the one-month follow-up survey, the average percentage of correct responses was 63.0%, with a similarly large effect size compared to baseline (Cohen *d* = 0.988), indicating sustained improvement over time. The small negative effect size between immediate and one-month follow-up (Cohen *d* = −0.253) suggests slight attenuation over time, although the overall effect remained substantial. Detailed item-level data are presented in Supplemental Table S1, and a concise summary of the most notable item-level changes is provided here to contextualize the overall score trends.

Across 17 items, several domains demonstrated statistically significant improvements. Notably, significant increases from baseline to immediate postintervention surveys were noted in the knowledge of hospital emission sources (Q10), sterilization hazards (Q12), and the relative magnitude of healthcare-associated pollution harm (Q15) (*P* < .001, *P* = .020, and *P* = .009, respectively), with gains in knowledge maintained at one month in the same three domains (*P* = .030, *P* = .010, and *P* = .040). Awareness of the global ranking of healthcare emissions (Q17) also improved significantly at one month compared with baseline (*P* = .040). A single item related to life-cycle analysis (Q13) showed a modest decline from immediate to one-month follow-up (*P* = .050). All other items demonstrated non-significant differences across time points (*P* > .050), indicating stable retention for most concepts.

### Attitude Results

Eight Likert-scale items (1 = strongly disagree to 5 = strongly agree) assessed residents’ attitudes toward sustainability in EM at baseline (N = 34) and immediately postintervention (n = 28). Median scores across all items ranged from 3 (“neutral”) to 4 (“agree”), reflecting generally neutral-to-positive attitudes prior to the intervention. When attitude items were averaged into a composite score, residents demonstrated a statistically significant improvement immediately postintervention (*P* = .001), as shown in Figure 2.

Despite this overall increase, item-level analyses did not show statistically significant changes, with most items exhibiting small directional shifts toward greater agreement, particularly regarding the importance of sustainability in clinical care and interest in further learning. Agreement with the negatively worded item assessing perceived impracticality of sustainability in the ED decreased slightly, indicating a modest shift toward lower agreement with the impracticality statement. These item-level findings are presented in Supplementary Table S2.

### Self-Reported Behavior Results

Three self-reported behavior items were assessed at baseline and at the one-month follow-up. A paired *t*-test comparing overall self-reported behavior scores demonstrated a statistically significant improvement from baseline to one month (*P* = .001) consistent with the differences illustrated in Figure 3.

Two items showed significant change. Residents were more likely to report that they would preferentially prescribe dry-powder inhalers over metered-dose inhalers at one month (12.0% versus 37.0%; *P* < .050), and significantly more residents reported actively trying to reduce unnecessary waste in their clinical practice (56.0% versus 87.0%; *P* < .050). No significant change was observed in preferences for prescribing a nebulizer over an inhaler.

For the open-ended item, thematic patterns remained stable. “Reduce” was the most common response at both time points (50.0% baseline; 60.0% one month), followed by smaller proportions referencing reuse strategies or describing tension between sustainability and patient care. The proportion of noninformative responses remained unchanged. All behavior results, including thematic frequencies, are summarized in Table 2.

## DISCUSSION

This study evaluated the impact of an educational intervention on EM residents’ knowledge, attitudes, and self-reported behaviors related to environmentally sustainable clinical practice. Sustainability remains largely absent from traditional EM training, despite healthcare’s substantial contribution to greenhouse gas emissions and growing national calls for climate-conscious clinical decision-making.^9^ Our findings demonstrate that residents’ attitudes toward sustainable practices, while already generally neutral to positive at baseline, showed a significant improvement immediately after the intervention. This attitudinal shift corresponded with meaningful changes in self-reported sustainability-related behaviors at one month. A low-burden, single-session module can meaningfully improve residents’ understanding of key sustainability concepts and influence an intention to change certain practice behaviors, even within the constraints of a busy residency curriculum.

Residents demonstrated meaningful improvements in sustainability-related knowledge immediately following the intervention, with overall retention at one month. The strongest gains occurred on question items addressing hospital emission sources, sterilization safety, and healthcare’s contribution to pollution, concepts that are rarely emphasized in EM training and are generally unfamiliar to clinicians.^4^ These findings suggest that brief, structured instruction can effectively close foundational knowledge gaps, particularly in domains lacking clinical exposure or experiential reinforcement. The question item addressing the understanding of Scope 3 emissions—the indirect, supply-chain emissions that make up most of the healthcare’s carbon footprint—also improved.^13^ In contrast, Scope 1 (direct emissions from onsite fuel use) and Scope 2 (indirect emissions from purchased electricity) represent a much smaller share of total healthcare emissions.^13^ Notably, increased awareness of the disproportionate impact of Scope 3 emissions is particularly relevant to everyday decision-making in the ED, where equipment, pharmaceuticals, and disposables contribute to the overall environmental impact. Knowledge gains that persisted at one month further support the durability of the intervention and reinforce the value of integrating sustainability content into formal resident education.

Residents’ attitudes toward sustainability were generally neutral to positive at baseline, typically 3–4 on a five-point Likert scale and demonstrated a statistically significant improvement immediately postintervention. Although the median Likert values shifted only modestly, the paired statistical comparison showed consistent upward movement across multiple attitude items, reflecting a meaningful attitudinal shift despite a relatively high baseline starting point. Such ceiling effects can partially mask visible changes and limit the measurable impact of a single educational exposure, particularly when learners already agree with the underlying concepts. Prior research consistently shows shifting attitudes to be more resistant to change than improving knowledge or self-reported behaviors, often requiring repeated reinforcement, hands-on experience, or institutional cues to produce measurable movement.^14^ The small directional shifts toward greater agreement, particularly regarding the importance of sustainable practice and the desire to learn more, may suggest ongoing receptivity and potential for cumulative change with repeated or more immersive educational touchpoints.

Self-reported behaviors were also evaluated in this study. Two of the three self-reported behavior items demonstrated significant improvement at one month, underscoring the practical impact of the intervention. Residents became more likely to preferentially prescribe dry-powder inhalers over metered-dose inhalers and more frequently reported actively attempting to reduce unnecessary waste in their clinical practice. These findings reflect shifts in clinical practice, highlighting the importance of education and its ability to translate into modifiable and actionable habits. The lack of change in nebulizer versus inhaler prescribing is unsurprising, as this decision is typically driven by patient acuity, airway dynamics, and institutional protocols rather than physician preference.^15^ Overall, the observed behavior changes suggest that brief, targeted education can influence day-to-day decision-making in the ED, even in the absence of broader structural or policy modifications.

These findings align with emerging literature demonstrating that targeted educational interventions can improve sustainability-related knowledge and clinical decision-making in other specialties, particularly anesthesia and perioperative care.^16^ Prior initiatives, such as the adoption of low–global warming potential anesthetic agents or waste-reduction programs in operating rooms, have similarly shown that clinicians respond positively to structured, evidence-based sustainability training.^17^ However, published data within EM remains limited. Existing literature consists largely of conceptual reviews, case examples, and proposed frameworks rather than evaluated interventions with measurable outcomes, as reflected in prior reviews identifying a gap in the EM evidence base and calling for further research to inform practice.^9^ By providing quantitative evidence that a brief, scalable module can meaningfully improve knowledge, enhance EM residents’ attitudes toward sustainable healthcare, and influence practice behaviors among them, this study fills an important gap and supports the feasibility of incorporating sustainability education into routine residency training.

The observed improvements in knowledge, attitude and the reported practice behaviors of residents highlight the feasibility of integrating sustainability content into EM education with minimal disruption to existing curricula. Because the intervention used freely available, asynchronous materials, it represents a low-cost and easily scalable approach that residency programs can adopt as part of didactics, onboarding, or annual competency training. The findings also underscore the potential for sustainability principles to become embedded within routine clinical decision-making when learners are provided with clear, actionable guidance. As hospitals increasingly aim to reduce their environmental footprint, aligning resident education with institutional sustainability initiatives may further reinforce behavior change and promote a consistent culture of resource stewardship across emergency care settings.

## LIMITATIONS

This study has several limitations. It was conducted within a single hospital system, which may limit generalizability to other EM programs with different resources, workflows, or institutional cultures. Participation was based on attendance at scheduled didactics, creating potential selection bias and modest sample sizes at each time point. Although response rates were modest, this is consistent with survey-based research among medical trainees. Importantly, response rate alone does not necessarily indicate nonresponse bias, as nonresponse bias depends on whether the characteristics or opinions of nonrespondents differ meaningfully from those of respondents. Prior work has demonstrated that response rate and nonresponse bias are related but distinct constructs and that low response rates do not inherently invalidate survey findings in medical education research.^18^ Sex-stratified analyses were not performed because differences by sex were not hypothesized; however, the study included both male and female residents.

Behavioral outcomes were self-reported and may not fully reflect objective practice patterns. The survey instrument was developed for this project and was not previously validated, which may limit the precision and interpretability of the knowledge, attitude, and behavior measures. Additionally, the one-month follow-up period limits insight into long-term retention or sustained self-reported behavior change. Furthermore, the intervention consisted of a single, standalone video module and a peer-reviewed paper without facilitated discussion or reinforcement, which may explain the modest improvement of attitude scores and highlight the need for more longitudinal curricula. Despite these limitations, the study provides early, quantitative evidence supporting the feasibility of sustainability-focused education in EM.

## CONCLUSION

A brief educational intervention was associated with improvements in emergency medicine residents’ knowledge of healthcare sustainability, strengthened their attitudes toward environmentally responsible clinical care, and influenced key self-reported clinical behaviors. These findings demonstrate that sustainability education can be feasibly integrated into existing residency training using low-cost, scalable materials. As healthcare systems increasingly pursue environmental accountability, incorporating structured sustainability curricula into EM training may help prepare future clinicians to make informed, environmentally conscious decisions in everyday practice. Future studies should evaluate multicenter implementation, objective measures of behavior change, and the impact of repeated or longitudinal educational exposure.

## Supplementary Information



## Figures and Tables

**Figure 1:**
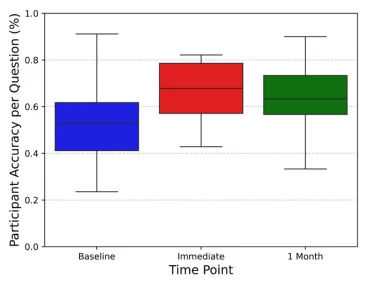
Overall score trends following a brief educational module on sustainable clinical practice delivered in two community-based emergency medicine residency programs.

**Figure 2:**
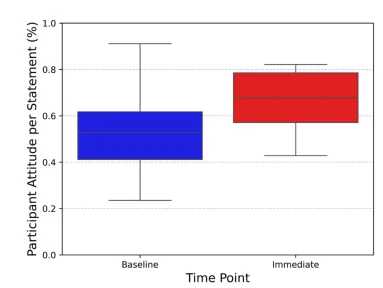
Shifts in perceptions of the importance, feasibility, and relevance of environmental sustainability in clinical practice among emergency medicine residents.

**Figure 3:**
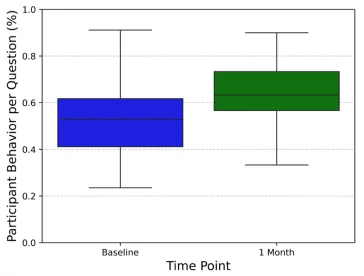
Changes in sustainability-related clinical behaviors following a brief educational intervention among 34 emergency medicine residents.

**Table 1:** Demographic characteristics of emergency medicine residents who completed the baseline survey before participating in a sustainability education intervention in a study of sustainability training.

Characteristic	Category	N = 34 (%)
PGY level	PGY-1	14 (41.2%)
	PGY-2	14 (41.2%)
	PGY-3	6 (17.6%)
Gender	Male	22 (64.7%)
	Female	12 (35.3%)
	Non-binary / prefer not to say	0 (0.0%)
Additional degrees	Yes	14 (41.2%)
	No	20 (58.8%)

*PGY*, postgraduate year.

**Table 2:** Item-level self-reported sustainability behaviors at baseline and one-month follow-up of emergency medicine residents after completing a sustainability education module.

Question	Behavior item	Baseline % (N = 34)	1-Month % (n = 30)	Significance
Q.1	Preferentially prescribe DPI over MDI (Yes)	12%	37%	*P* < .050
Q.2	Preferentially prescribe nebulizer over inhaler (Yes)	26%	40%	ns
Q.3	Actively try to reduce unnecessary waste (Yes)	56%	87%	*P* < .050
Q.4	Sustainability practices (open-ended)			
	• Reduce	50%	60%	n.s.
	• Reuse/Recycle	12%	7%	
	• Tension/Impact on patient care	9%	7%	
	• Not Informative	29%	33%	

*DPI*, dry-powder inhaler; *MDI*, metered-dose inhaler; *ns*, nonsignificant.
