# Supplementary material for: Evaluating the Impact of a Sustainability Education Intervention on Emergency Medicine Residents
Source: West J Emerg Med. 2026 Aug 10;27(5):1118–25. doi: 10.5811/westjem.56998 (PMC13660593; doi:10.5811/westjem.56998)
Supplement: Supplementary file 1 [file wjem-27-1118-s001.docx]

**Supplementary Materials:**

Table S1. Item-Level Knowledge Assessment Scores and Pairwise Comparisons in an Educational Intervention on Sustainable Clinical Practice Among Emergency Medicine Residents.

| **Q#** | **Full Question Content** | **Correct Response** | **Baseline (%)** | **Immediate (%)** | **1-Month (%)** | **p (Base→**  **Immediate)** | **p (Base→1M)** | **p (Immediate→1M)** |
| --- | --- | --- | --- | --- | --- | --- | --- | --- |
| Q1 | Reusable PPE always has lower environmental impact than single-use PPE. | False | 41 | 57 | 43 | n.s. | n.s. | n.s. |
| Q2 | One puff of an MDI ≈ driving a car one mile. | True | 50 | 71 | 73 | n.s. | n.s. | n.s. |
| Q3 | Semicritical devices require high-level disinfection. | True | 91 | 79 | 90 | n.s. | n.s. | n.s. |
| Q4 | Generic Proventil has less GWP than generic Ventolin. | False | 38 | 43 | 33 | n.s. | n.s. | n.s. |
| Q5 | DPI has lower GWP than MDI. | True | 59 | 79 | 80 | n.s. | n.s. | n.s. |
| Q6 | DPI results in worse outcomes than MDI. | False | 35 | 32 | 30 | n.s. | n.s. | n.s. |
| Q7 | Maximizing SABA use lowers GWP. | False | 44 | 61 | 53 | n.s. | n.s. | n.s. |
| Q8 | Hospital quality metrics prioritize carbon footprint over patient safety. | False | 62 | 75 | 63 | n.s. | n.s. | n.s. |
| Q9 | Lower carbon footprint: nebulizer or inhaler? | Inhaler | 53 | 57 | 60 | n.s. | n.s. | n.s. |
| **Q10** | Category contributing most to hospital carbon footprint? | Scope 3 | 44 | 82 | 70 | **0.002** | **0.030** | n.s. |
| Q11 | PPE factor causing infection control vs sustainability tension. | Single-use PPE increases waste but reduces infection risk | 68 | 79 | 83 | n.s. | n.s. | n.s. |
| **Q12** | Sterilization method with occupational safety concerns. | Ethylene oxide | 29 | 57 | 57 | **0.030** | **0.010** | n.s. |
| **Q13** | Approach balancing reusable vs disposable devices. | Risk stratification + life-cycle analysis | 62 | 82 | 57 | n.s. | n.s. | **0.050** |
| Q14 | Factor reducing low-value care + environmental impact. | Improving chronic care & ACP | 62 | 68 | 77 | n.s. | n.s. | n.s. |
| **Q15** | Pollution harm vs medical errors. | Same order of magnitude | 24 | 57 | 50 | **<0.010** | **0.040** | n.s. |
| Q16 | % of U.S. GHG emissions attributable to healthcare. | 8.5% | 68 | 61 | 70 | n.s. | n.s. | n.s. |
| **Q17** | If healthcare were a country, it would be the ___ largest emitter. | 5th | 35 | 61 | 63 | n.s. | **0.040** | n.s. |

*This table summarizes the percentage of residents answering each knowledge item correctly at baseline (n=34), immediately post-intervention (n=28), and one-month follow-up (n=30), along with pairwise statistical comparisons. Abbreviations: MDI, metered-dose inhaler; DPI, dry-powder inhaler; GWP, global warming potential; PPE, personal protective equipment; n.s., not significant.*

*TableS2. Item-Level Attitude Responses Toward Sustainable Clinical Practice Among Emergency Medicine Residents Before and After an Educational Intervention.*

| **Question** | **Attitude Statement** | **Baseline Median (IQR)** | **Immediate Median (IQR)** | **Direction of Change** |
| --- | --- | --- | --- | --- |
| Q1 | Environmental sustainability should be a high priority during routine patient care. | 3 (2–4) | 4 (3–4) | ↑ Toward Agreement |
| Q2 | Sustainability can be achieved without increasing infectious risk to patients. | 3 (2–3) | 3.5 (3–4) | ↑ Toward Agreement |
| Q3 | The emergency department can make meaningful contributions to reducing healthcare’s environmental impact. | 3 (2–4) | 4 (3–4) | ↑ Toward Agreement |
| Q4 | Incorporating sustainability will not compromise patient care. | 3 (2–4) | 3 (3–4) | Stable |
| Q5 | Sustainability should be included in emergency medicine residency training. | 3 (2–4) | 3 (3–4) | Stable |
| Q6 | Emergency physicians should consider sustainability as part of quality patient care. | 3 (2–4) | 3 (3–4) | Stable |
| Q7 | I am interested in learning more about how to integrate sustainability into emergency medicine. | 3 (2–4) | 4 (3–4) | ↑ Toward Agreement |
| Q8* | It is unrealistic to expect emergency departments to become truly sustainable. | 3 (2–4) | 2.5 (2–3) | ↓ (Less Agreement) |

*****Negatively worded item.

*Median Likert-scale responses (1=strongly disagree to 5=strongly agree) at baseline (n=34) and immediately post-intervention (n=28), with directional shifts indicating changes in attitudes. Abbreviations: IQR, interquartile range.*
